# Participation in Virtual Urology Conferences During the COVID-19 Pandemic: Cross-sectional Survey Study

**DOI:** 10.2196/24369

**Published:** 2021-04-21

**Authors:** Menghua Wang, Banghua Liao, Zhongyu Jian, Xi Jin, Liyuan Xiang, Chi Yuan, Hong Li, Kunjie Wang

**Affiliations:** 1 Department of Urology Institute of Urology (Laboratory of Reconstructive Urology) West China Hospital, Sichuan University Chengdu China

**Keywords:** virtual conference, COVID-19, survey

## Abstract

**Background:**

Due to the influence of the COVID-19 pandemic, conventional face-to-face academic conferences have been restricted, and many of these conferences have moved onto the internet.

**Objective:**

The aim of this study was to investigate the virtual conferences in the field of urology during the COVID-19 pandemic and provide suggestions for better organization of such conferences.

**Methods:**

A cross-sectional survey was conducted from May 30 to June 15, 2020, in China. Our team designed a 23-item questionnaire to investigate the conferences attended by urologists during the COVID-19 pandemic. SPSS 22.0 (IBM Corporation) was applied to analyze the data collected.

**Results:**

A total of 330 Chinese urologists participated in our survey, and the response rate was 89.7% (330/368). Among the participants, 40.9% (135/330) were associate chief physicians. The proportion of participants who took part in conventional face-to-face academic conferences decreased from 92.7% (306/330) before the COVID-19 pandemic to 22.1% (73/330) during the pandemic (*P*<.001). In contrast, the proportion of urologists who took part in virtual conferences increased from 69.4% (229/330) to 90% (297/330) (*P*<.001). Most urologists (70.7%, 210/297) chose to participate in the virtual conferences at home and thought that a meeting length of 1-2 hours was most appropriate. Among the urologists, 73.7% (219/297) reported that their participation in the virtual conferences went smoothly, while the remaining respondents reported that they had experienced lags in video and audio streaming during the virtual conferences. When comparing conventional face-to-face conferences with virtual conferences, 70.7% (210/297) of the respondents thought that both conference formats were acceptable, while 17.9% (53/297) preferred virtual conferences and 11.5% (34/297) preferred conventional face-to-face meetings.

**Conclusions:**

Virtual conferences are increasing in popularity during the COVID-19 pandemic; however, many aspects of these conferences could be improved for better organization.

## Introduction

Since the first case of COVID-19 was reported in Wuhan, China, the disease has spread worldwide rapidly [1]. To prevent the spread of COVID-19, many mass gathering events have been canceled or postponed, including the annual meetings of the European Association of Urology (EAU) and the American Urological Association [2,3]. In this condition, the role of virtual conferences is expanding [4], and many academic conferences moved onto the internet, such as the EAU20 Virtual Congress [5].

However, to our knowledge, there is no study investigating virtual conferences in the field of urology. In this study, we aimed to investigate the virtual meetings during the COVID-19 pandemic in China and provide advice on organizing efficient and high-quality virtual conferences according to the results of our survey.

## Methods

Our team designed a structured questionnaire after reviewing the current relevant literature. A total of 23 items were finalized for this survey, covering demographics (2 items), traditional academic conferences (5 items), virtual (video) conferences (11 items), comparison between traditional and virtual conferences (3 items), and topics of academic conferences (2 items). We have provided the questionnaire in Multimedia Appendix 1.

We collected the data through the *Wenjuanxing* website [6] from May 30 to June 15, 2020. All participants accessed the questionnaire by clicking on the survey link. Participants were able to submit the questionnaires only when all the items were completed. To avoid repetition, IP restriction was applied, which meant that the survey could only be completed once from a single IP address.

Data are presented as number (percentage) for categorical variables, and chi-square tests were used to compare the distributions of categorical variables. All the statistical analyses were conducted using SPSS 20.0 (IBM Corporation).

## Results

From May 30 to June 15, 2020, we sent the web-based questionnaire to 368 Chinese urologists; 330 of them completed the survey, resulting in a response rate of 89.7%. Among the participants, 96.4% (318/330) were based at secondary grade A hospitals and above. Regarding their academic titles, most of the participants were associate chief physicians (135/330, 40.9%), followed by attending physicians (113/330, 34.2%), chief physicians (61/330, 18.5%), and residents (21/330, 6.4%) (Table 1).

**Table 1:** Baseline information about the survey participants (N=330).

Characteristic		Value, n (%)
**Hospital level^a^**		
	Tertiary grade A	168 (50.9)
	Tertiary grade B	97 (29.4)
	Secondary grade A	53 (16.1)
	Secondary grade B	6 (1.8)
	Primary and others	6 (1.8)
**Academic title^b^**		
	Chief physician	61 (18.5)
	Associate chief physician	135 (40.9)
	Attending physician	113 (34.2)
	Resident	21 (6.4)

^a^In China, hospitals are classified as primary, secondary, or tertiary institutions. Typically, primary hospitals contain less than 100 beds and secondary hospitals contain 100-500 beds, while tertiary hospitals have a bed capacity exceeding 500. According to their medical quality, etc, each hospital level is further subdivided into three subsidiary grades of A, B, and C, which results in a total of 9 levels.

^b^For the physicians’ hierarchy in China, there are four levels in total, namely chief physician, associate chief physician, attending physician, and resident, from senior to junior.

The proportion of urologists participating in conventional face-to-face conferences decreased from 92.7% (306/330) before the COVID-19 pandemic to 22.1% (73/330) during the pandemic (*P*<.001). Generally, 89.5% (274/306) of the urologists were satisfied with the traditional conferences, while others (32/306, 10.5%) thought that live conferences were time-consuming (27/32, 84%,) and expensive (20/32, 62.5%).

The proportion of urologists participating in virtual conferences increased from 69.4% (229/330) before the COVID-19 pandemic to 90% (297/330) during the pandemic (*P*<.001). Of the participants, 99.0% declared that they were “very satisfied” or “satisfied” with the virtual conferences.

Most of the urologists (70.7%, 210/297) chose to take part in the virtual conferences at home. In terms of the length of the virtual conferences, most participants (62.3%, 185/297) reported that they usually spent 0.5-1.5 hours per meeting, and 66.3% (197/297) thought that 1-2 hours is the most appropriate duration for a meeting.

Regarding the software, Tencent Meeting, DingTalk, and Zoom were the most commonly used platforms in China. Of the urologists, 73.7% (219/297) reported that their participation in the virtual meetings went smoothly, while the remaining urologists said that due to limited bandwidth or unstable web-based platforms, they experienced lags in video and audio streaming during the virtual conferences. According to our survey, the urologists thought that a good web-based platform should provide on-demand video and should be stable and interactive.

When comparing conventional face-to-face conferences with virtual conferences, 70.7% (210/297) of the participants thought that both formats were acceptable, while 17.9% (53/297) preferred virtual conferences and 11.5% (34/297) preferred conventional face-to-face meetings. Compared with conventional face-to-face meetings, the urologists reported that virtual conferences were time-effective, less expensive, and more convenient; however, they also lacked interaction, body language, and Continuing Medical Education (CME) credits.

**Figure 1:**
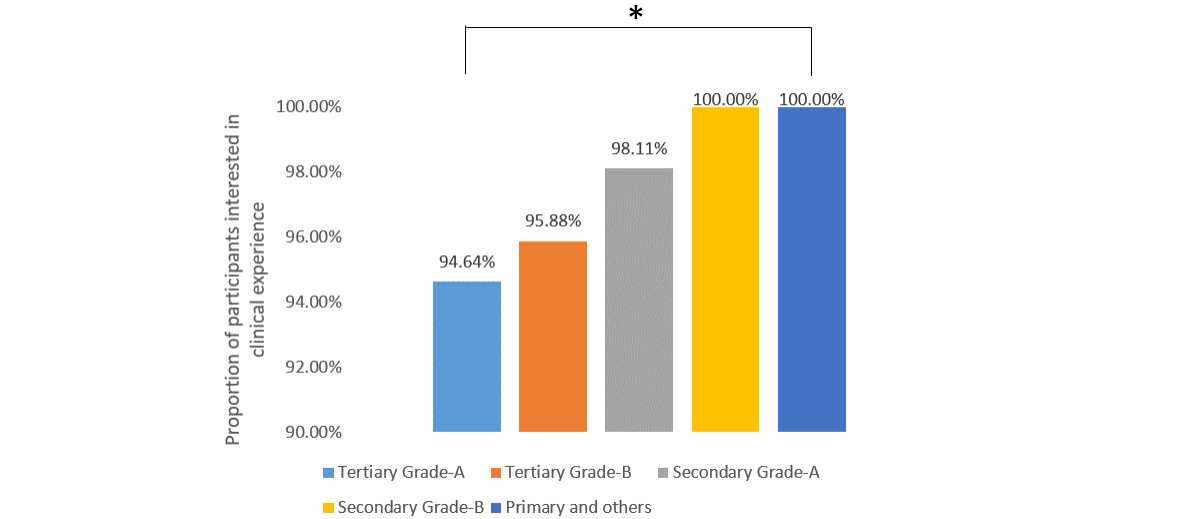
Proportions of participants interested in learning about clinical experience at urology conferences stratified by hospital level. *P＞.05.

**Figure 2:**
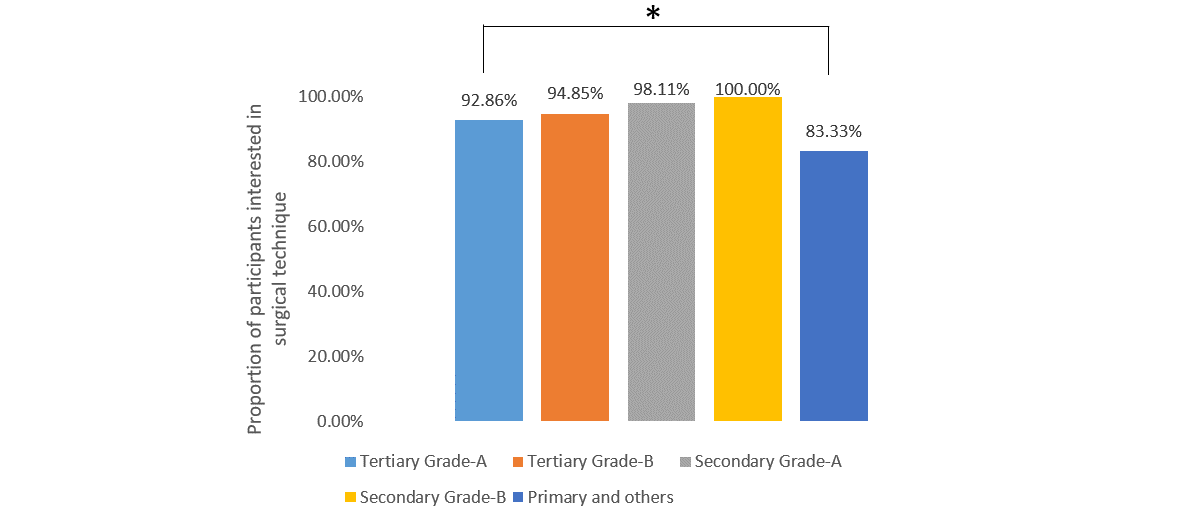
Proportions of participants interested in learning about surgical technique at urology conferences stratified by hospital level.*P＞.05.

**Figure 3:**
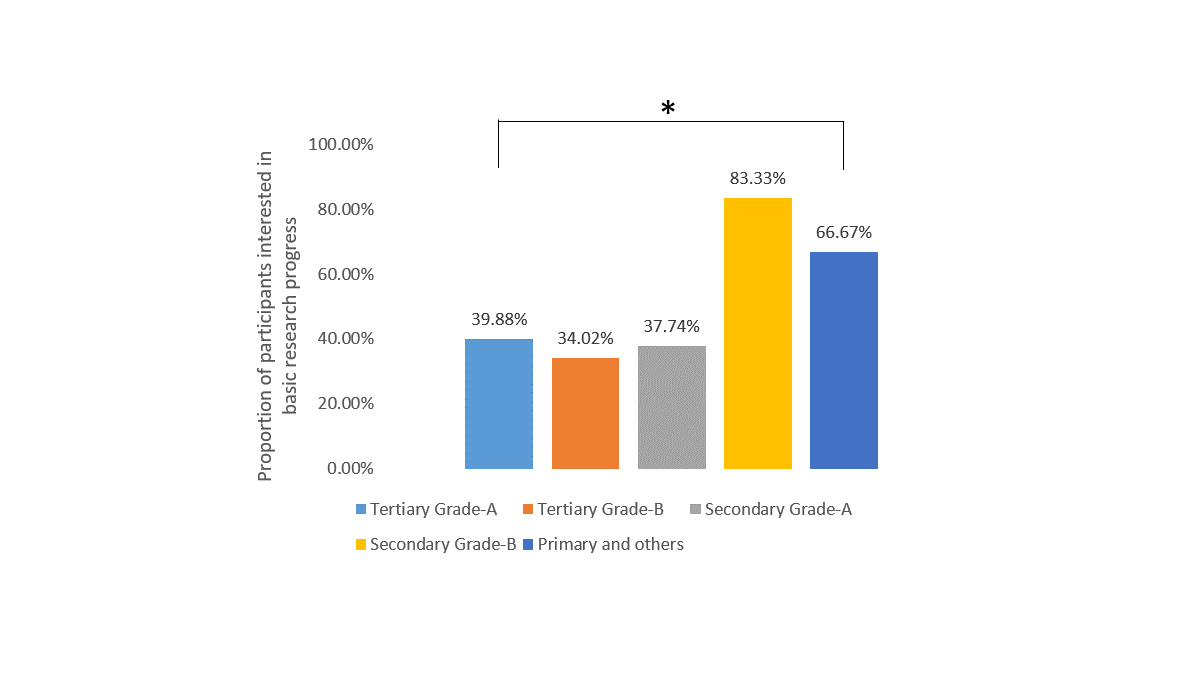
Proportions of participants interested in learning about basic research progress at urology conferences stratified by hospital level.*P＞.05.

**Figure 4:**
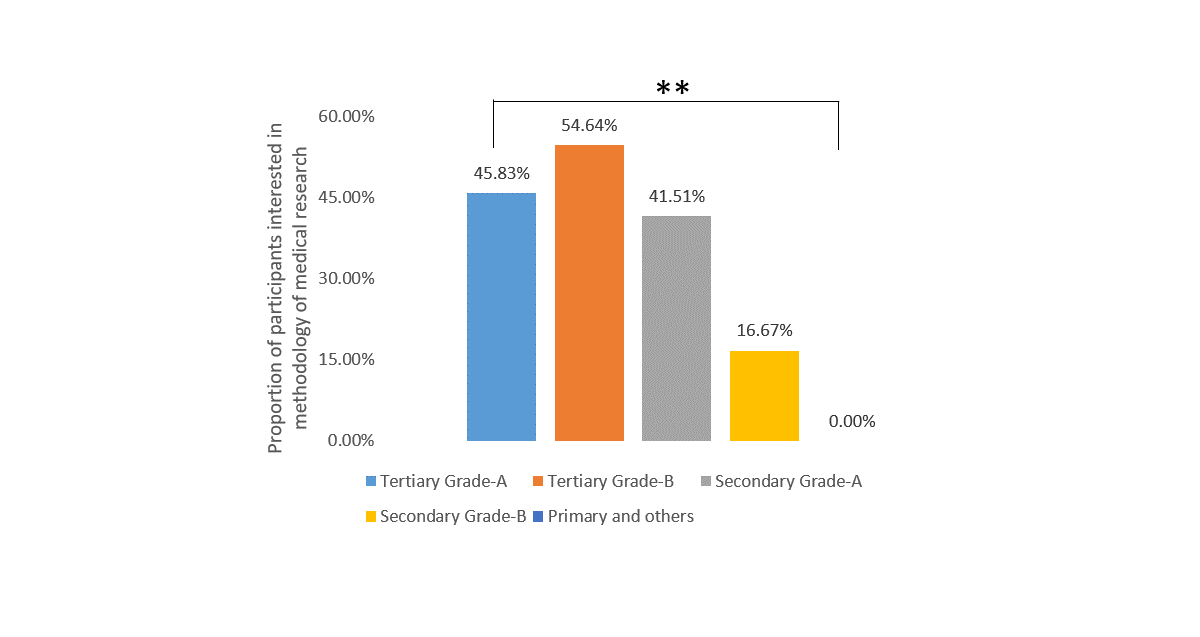
Proportions of participants interested in learning about the methodology of medical research at urology conferences stratified by hospital level. **P＜.05.

For the different topics of urological conferences, stones (286/330, 86.7%), laparoscopy (84.9%, 280/330), urological oncology (84.6%, 279/330), and prostatic diseases (74.9%, 247/330) were the four most popular topics, followed by andrology (177/330, 53.6%), female urology (140/330, 42.4%), reconstructive urology (130/330, 39.4%), and kidney transplantation (17/330, 5.2%). When the participants were asked about what they expected to learn about during urological conferences, clinical experience and surgical techniques were selected by 95.8% (316/330) and 94.2% (311/330) of participants, while the methodology of medical research and basic research progress were only chosen by 46.4% (153/330) and 39.1% (129/330) of the participants. Further analysis showed that there was no significant difference among the different levels of hospitals for interest in clinical experience (Figure 1), surgical technique (Figure 2), or basic research progress (Figure 3), while fewer urologists based at secondary grade A hospitals and below were interested in the methodology of medical research (Figure 4).

## Discussion

### Principal Findings

As far as we know, this is the first survey to investigate urological conferences during the COVID-19 pandemic. A total of 330 Chinese urologists, with different academic titles and from different levels of hospitals, were included in our survey. Our study revealed that although virtual conferences are increasing in popularity during the COVID-19 pandemic, the organization of these virtual conferences needs further improvement.

During the COVID-19 pandemic, traditional face-to-face meetings have been restricted, and it is more important than ever to maximize virtual meetings. In our survey, the proportion of urologists who took part in virtual conferences increased from 69.4% before the COVID-19 pandemic to 90.0% during the pandemic, indicating a sharp increase in virtual conference participation. Additionally, due to the development of efficient telecommunication networks, technological capabilities are no longer a hindrance, and these networks have been used in various areas [7,8]. Recently, the Human Genome Meeting, Global Genomic Medicine Collaborative, and Trans Tasman Radiation Oncology Group Annual Scientific Meeting were delivered virtually and achieved great success, with 97% of the participants being “very satisfied or satisfied” with the virtual style [9,10]; this was similar to our 99.0% proportion of satisfied participants, indicating that virtual conferences were well accepted by urologists. As a result, we should maximize the use of virtual conferences to ensure communication in the medical community during the COVID-19 pandemic.

When preparing for virtual conferences, the first task is to define the target audience, as different audiences may have different appetites. For example, in our study, the urologists who worked at secondary grade A hospitals and below had lower enthusiasm for the topic of medical research methodology. Second, according to our survey, most people chose to take part in conferences at home; therefore, avoiding early morning and late evening meetings would provide them with a better experience [11]. Web-based platform selection is another essential aspect of the success of virtual conferences. In our survey, Tencent Meeting, DingTalk, and Zoom were the most commonly used platforms. In addition to these platforms, webinar technology [12], Twitter [13], journal clubs, podcasts, *Intouch Vita*, etc, were used for virtual meetings [14]. However, regardless of which platform is chosen, it should ensure a smooth and stable video experience; this was what people were most concerned about according to our survey.

During virtual conferences, the duration of the sessions should be controlled, as the average attention span for adults is only approximately 15-20 minutes [15]. In our study, most of the urologists thought that 1-2 hours was the most appropriate length. Salomon and their team [16] designed each session at their virtual conference to last 2 hours, and this schedule was appreciated by most of the participants (135/181, 74.6%), which was in accordance with our respondents’ preference of 1-2 hours. As a result, the duration of an academic meeting should be controlled within approximately 2 hours. Additionally, as mentioned before, one of the limitations of virtual conferences is the lack of interaction. To solve this problem, organizers could increase the time allotted to question-and-answer periods [10], permit attendees to turn on their cameras, provide virtual open discussion chat rooms [16], etc.

When a virtual conference has ended, the organizer should provide on-demand content such as video on the virtual platform. On-demand video is important for people in different time zones and is valuable for all participants to catch up on missed talks. Over 90% of the urologists in our survey thought that on-demand video was essential for their learning. Additionally, the physicians who attend virtual conferences should be provided with CME credits. In the era of information explosion, to enable physicians to remain current with their medical knowledge, CME has been implemented in many countries worldwide, and a variable number of CME credits is required [17,18]. Normally, CME credits can be earned through attendance at in-person academic conferences, workshops, symposiums, etc. However, according to our survey, some urologists reported that during virtual conferences, CME credits were not made available to participants, which in our view may reduce the enthusiasm of urologists to take part in these conferences. As a result, in the future, we believe that virtual conferences should also provide CME credits.

In terms of the comparison between virtual conferences and conventional face-to-face conferences, as mentioned before, both formats have their advantages and disadvantages. As a result, some researchers have proposed a new type of meeting called a hybrid meeting, which combines aspects of virtual and live conferences. In a survey conducted by Forrest et al [10], 85% of the delegates reported that they preferred hybrid meetings compared with virtual or physical meetings only. Additionally, Gomez Rivas et al [19] shared their preliminary experience of hybrid meetings that included both onsite and web-based participants. Moreover, we believe that this type of meeting would be the best choice for academic conferences in the future, as it combines the advantages of both formats.

### Limitations

There are several limitations to our survey. First, our survey had a relatively small sample size. Second, most of the participants in this study were from tertiary hospitals. Despite these two limitations, our study provides a preliminary report of virtual conferences during the COVID-19 pandemic and provides many suggestions on how to organize an efficient and satisfying virtual conference, which is timely and useful.

### Conclusion

Virtual conferences are increasing in popularity during the COVID-19 pandemic; however, many aspects of these conferences could be improved for better organization.

## Appendix

Multimedia Appendix 1 Detailed questionnaire.

## Abbreviations

CME: Continuing Medical Education
EAU: European Association of Urology
