# Supplementary material for: Participation in Virtual Urology Conferences During the COVID-19 Pandemic: Cross-sectional Survey Study
Source: J Med Internet Res. 2021 Apr 21;23(4):e24369. doi: 10.2196/24369 (PMC8061892; doi:10.2196/24369)
Supplement: Multimedia Appendix 1 [file jmir_v23i4e24369_app1.docx]

**Supplementary material**

**Demographics**

1. ***What’s the level of your hospital?***

| Tertiary Grade-A |
| --- |
| Tertiary Grade-B |
| Secondary Grade-A |
| Secondary Grade-B |
| Primary and others |

1. ***What’s your academic title?***

Chief Doctor

Associate Chief Doctor

Attending Doctor

Resident Doctor

**Conventional face-to-face academic conferences**

1. ***Have you ever participated in conventional face-to-face academic conferences before this COVID-19 pandemic?***

Yes

No

1. ***How often did you participate in conventional face-to-face academic conferences before this COVID-19 pandemic?***

1 time/month

2 times/month

3-4 times/month

＞4 times/month

1. ***Have you ever participated in conventional face-to-face academic conferences during this COVID-19 pandemic?***

Yes

No

1. ***Are you satisfied with current conventional face-to-face academic conferences?***

Yes

No

1. ***Why are you unsatisfied with the current conventional face-to-face academic conferences? (Allow multiple answers)***

It’s time-consuming

It’s expensive

Other (please specify)

**Virtual academic conferences**

1. ***Have you ever participated in virtual academic conferences before this COVID-19 pandemic?***

Yes

No

1. ***How often do you participate in virtual academic conferences before this COVID-19 pandemic?***

＜1 time/month

1 time/month

2 times/month

≥3times/month

1. ***Have you ever participated in virtual academic conferences during this COVID-19 pandemic?***

Yes

No

1. ***How often do you participate in virtual academic conferences during this COVID-19 pandemic?***

1-2 times/month

3-5 times/month

6-8 times/ month

≥9 times/month

1. ***Are you satisfied with current virtual academic conferences?***

Very satisfied

Satisfied

Unsatisfied

1. ***Where do you usually participate in virtual academic conferences?***

Home

Hospitals

Other (please verify)

1. ***How long do you usually spend on the virtual academic conferences each time?***

≤0.5 hour

0.5-1.5 hours

1.5-2.5 hours

2.5-3.5 hours

＞3.5 hours

1. ***How long do you think is most appropriate for each virtual academic conference?***
   1. hour

1-2 hours

2-3 hours

＞3 hours

1. ***Which of the following online platforms have you used? (Allow multiple answers)***

Tencent Meeting

DingTalk

Zoom

Other (please verify)

1. ***Can you participate in the online virtual academic conferences smoothly?***

Yes, the quality of network is well

No, I often experience lags in video

1. ***What functions should the online platforms have? (Allow multiple answers)***

Stable

Interactive

Provide on-demand content

Other (please verify)

**Comparison between traditional and virtual academic conferences**

1. ***Comparing conventional academic conferences with virtual academic conferences, which one do you prefer?***

Conventional academic conferences

Virtual academic conferences

Both of them are acceptable

1. ***Comparing with conventional face-to-face academic conferences, what’s the advantages of virtual academic conferences?***

Time-effective

Cheaper

More convenient

Other (please verify)

1. ***Comparing with conventional face-to-face academic conferences, what’s the disadvantages of virtual academic conferences?***

Lack of interaction

Lack of body language

Lack of continuing medical education edits

Other (please verify)

**Topics of academic conferences**

1. ***What topics of academic conferences are you interested? (Allow multiple answers)***

Stones

Laparoscopy

Urological oncology

Prostatic diseases

Andrology

Female urology

Reconstructive urology

Kidney transplantation

1. ***What are you expected to learn during the academic conferences? (Allow multiple answers)***

Clinical experience

Surgical technique

Basic research progress

Methodology of medical search
